# Low-Cost Clamp-On Photometers (ClampOD) and Tube Photometers (TubeOD) for Online Cell Density Determination

**DOI:** 10.3389/fmicb.2021.790576

**Published:** 2022-01-13

**Authors:** Jörg S. Deutzmann, Grace Callander, Wenyu Gu, Albert L. Müller, Alexandra L. McCully, Jenna Kim Ahn, Frauke Kracke, Alfred M. Spormann

**Affiliations:** ^1^Civil and Environmental Engineering, Stanford University, Stanford, CA, United States; ^2^Chemical Engineering, Stanford University, Stanford, CA, United States

**Keywords:** photometer, continuous cell density measurement, online growth recording, anaerobic growth monitoring, online thermophilic growth monitoring, low-cost

## Abstract

Optical density (OD) measurement is the gold standard to estimate microbial cell density in aqueous systems. Recording microbial growth curves is essential to assess substrate utilization, gauge sensitivity to inhibitors or toxins, or determine the perfect sampling point. Manual sampling for cuvette-photometer-based measurements can cause disturbances and impact growth, especially for strictly anaerobic or thermophilic microbes. For slow growing microbes, manual sampling can cause data gaps that complicate analysis. Online OD measurement systems provide a solution, but are often expensive and ill-suited for applications such as monitoring microbial growth in custom or larger anaerobic vessels. Furthermore, growth measurements of thermophilic cultures are limited by the heat sensitivity of complex electronics. Here, we present two simple, low-cost, self-assembled photometers—a “TubeOD” for online measurement of anaerobic and thermophilic cultures in Hungate tubes and a “ClampOD” that can be attached to virtually any transparent growth vessel. Both OD-meters can be calibrated in minutes. We detail the manufacturing and calibration procedure and demonstrate continuous acquisition of high quality cell density data of a variety of microbes, including strict anaerobes, a thermophile, and gas-utilizing strains in various glassware. When calibrated and operated within their detection limits (ca. 0.3–90% of the photosensor voltage range), these self-build OD-meters can be used for continuous measurement of microbial growth in a variety of applications, thereby, simplifying and enhancing everyday lab operations.

## Introduction

Determination of suspended cell density in a liquid culture is essential in virtually any field of microbiology. In particular, accurate determination of microbial growth rate, cell yields, and growth phases are essential for basic microbial physiology, biotechnological applications, and clinical microbiology. Optical cell density measurements based on light scattering of suspended cells, often referred to as optical density (OD) or turbidity measurements, are the most widely used means to quantify cell mass in liquid cultures.

OD measurements estimate cell density based on the loss in intensity when light passes through a sample. OD is defined as a logarithmic decrease in light transmission (T), or OD = log_10_ (1/T). When light passes through a sample containing particles —including microbes— the liquid appears turbid because light is scattered. Therefore, turbidity of the sample can be measured as the decrease in light transmission through a sample when other modes of light attenuation, such as absorption by colored substances, are absent (Koch, 1968). Absorption (A) measurements are based on the Beer-Lambert law (*A* = ε⋅l⋅c), and are used to determine the concentration (c) of a substance of known absorptivity (ε) based on the transmitted light through a sample of length l. Light scattering by microorganisms has been studied in detail and differs from absorption measurements in several aspects (Koch, 1968, 1970; Koch and Ehrenfeld, 1968). (1) Microbes scatter light mainly at small angles (Koch, 1968), which causes OD measurements to vary depending on the geometry of the photometer (specifically the distance of sample and photosensor, in particular when the light beam is not narrow) (Koch, 2007). (2) Contrary to absorption measurements, light scattering by particles only follows the Beer–Lambert law (Swinehart, 1962) at low densities where the probability of light interacting with more than one particle on the light path is low and the particles (in this case, microbial cells) do not interact with each other (Koch, 2007). At low turbidity where the Beer-Lambert law is valid (e.g., *OD* < 0.3 for most photometers and microbial cultures at 600 nm wavelength), light attenuation is proportional to cell density and the path-length of the light through the turbid solution. It has been shown that this linear relationship holds true for OD and cell density (defined as cell dry weight per volume) over a variety of cell sizes and shapes (Koch, 2007). However, the correlation of cell count per volume and OD depends greatly on these properties and calibration for each strain, medium, and growth condition (Koch, 2007; Stevenson et al., 2016) is necessary.

At higher turbidities, this linear relationship between cell density and OD does not apply. One commonly used approach to acquire accurate cell density data is dilution of the turbid cell suspension until the turbidity of the diluted sample is in the linear range (Koch, 2007). However, the same publication also stated that at low cell densities, and, therefore, at low signal, errors introduced by variations between cuvettes, placement of the cuvette in the photometer, or pipetting and dilution errors have a relatively large impact that can exceed the error introduced by using a non-linear, parabolic calibration (Koch, 2007). For OD measurements exceeding the linear range, the relationship between OD and cell density can be approximated by a polynomial fit of second order to obtain accurate results (Koch, 1970, 2007).

In microbiological studies, OD is often measured at a wavelength between 540 and 660 nm, although shorter wavelengths enable more sensitive measurements (Koch, 1961). Mostly, OD is measured at 600 nm, because this wavelength is not harmful to microbes, and cheap filters for single wavelengths in this range became available in the 1940s and 50s. This wavelength range is also among the least absorbed by yellowish colored culture media containing yeast extract or amino acid mixes or by most pigmented microbial cultures (Koch, 2007; Stevenson et al., 2016). In laboratory settings, OD measurements are often performed by manually taking a sample from the microbial culture into a cuvette and measuring OD in a benchtop (spectro)photometer. However, when time-course data of microbial growth at variable kinetics are needed, discontinuous manual sampling often results in significant volume change, can introduce growth disturbances in anaerobic or thermophilic cultures by introducing traces of oxygen or causing temperature shocks, respectively. In addition, manual sampling limits data acquisition times and the amount of data points that can be collected. Often, conditions must be optimized over several iterations until a publication-quality growth curve can be obtained.

Automation of OD measurements is currently only available for limited experimental designs. Real-time OD measurements and data logging for aerobic microbes utilizing soluble substrates (or gases contained in ambient air) are easily automated and multiplexed using multi-well plates and plate readers (Stevenson et al., 2016). However, this method is not applicable for cultures grown in a variety of other culture vessels, such as small scale chemostats, electrochemical H-cells, shake flasks, conical plastic centrifuge tubes, etc., where monitoring growth is usually only possible by discontinuous manual sampling. In addition, automation of OD measurements for strict anaerobes and thermophiles has remained a challenge. Maintaining strictly anaerobic conditions, while quantifying gaseous substrates or products, requires incubation in gas-tight, closed incubation vessels, such as Hungate tubes, serum vials, or closed medium bottles (Widdel and Bak, 1992). A few research groups have developed and published promising online OD measurement devices for tubes using 3D printed tube holders (Lamb et al., 2013; Maia et al., 2016; Kutschera and Lamb, 2018; Sasidharan et al., 2018; Mauerhofer et al., 2019; Yallapragada et al., 2019), or a fitness-tracker based device with the promise to be attachable to a variety of culture vessels (Yallapragada et al., 2019). However, these devices rely on access to a 3D printer (Maia et al., 2016; Kutschera and Lamb, 2018; Sasidharan et al., 2018; Yallapragada et al., 2019) or availability of discontinued parts (Yallapragada et al., 2019). Further, the heat sensitivity of many electronic devices inhibits their use for online measurements of thermophilic microbes.

To tackle these issues, we developed a low-tech and affordable, clamp-on photometer (“ClampOD”) that can be attached to many different culture vessels and can be manufactured from readily available parts within a day. In addition, we developed a heat-stable low-tech tube photometer (“TubeOD”) out of the necessity to measure growth curves of thermophilic strains at 65°C. Here, we report the assembly, performance, and limitations of these devices, and show online acquisition of high-quality growth curves of a variety of microbes, including strict anaerobes, a thermophile, and a gas-utilizing anaerobe in different culture vessels.

## Materials and Equipment

### Materials and Equipment Used to Fabricate a ClampOD (Essential Parts: $13)

• Soldering station or conductive glue (e.g., silver epoxy)
• Pliers
• Wire stripper (or scissors)
• Screwdriver
• Parts
  ⚬ LED (C503B-AAN-CY0B0251, CREE LED, $0.15 at Newark.com)
  ⚬ LED driver (NSI45020AT1G, ON SEMICONDUCTOR, $0.52 at Newark.com)
  ⚬ Photosensor (TEMT6000, B07JB5TQ93, Comidox, $8.00 at Amazon.com for 4)
  ⚬ Multiconductor cable (24-14440, PRO POWER, $28.99 at Newark.com for 300 ft)
  ⚬ 5 min Epoxy (14250, Devcon, $10.35 at Grainger.com)
  ⚬ (optional) shrink tubing (HS101-1/16, Insultab, $3.03 at McMaster-Carr, Item 7496K82)
  ⚬ Connector receptacle 3 position (22-01-3037, MOLEX, $0.08 at Newark.com)
  ⚬ Connector headers 3 position (22-27-2031, MOLEX, $0.19 at Newark.com)
  ⚬ Contact sockets (08-50-0032, MOLEX, $0.18 at Newark.com)
  ⚬ Clamp (W001411A, HANGZHOU GREATSTAR INDUSTRIAL CO.LTD., $39.99 at Amazon.com for 6)
  ⚬ O-ring (0.070” dash number 009, $6.63 for 100 at McMaster-Carr, Item 9464K14)
  ⚬ (optional) matte tape (Magic Tape, Scotch, $3.50 at Amazon.com)

### Materials and Equipment Used to Fabricate a TubeOD (Essential Parts: $7.50)

• Soldering station or conductive glue (e.g., silver epoxy)
• Pliers
• Wire stripper (or scissors)
• Razor blade
• Cork borer (Humbold 3276G40)
• Parts
  ⚬ LED (C503B-AAN-CY0B0251, CREE LED, $0.15 at Newark.com)
  ⚬ LED driver (NSI45020AT1G, ON SEMICONDUCTOR, $0.52 at Newark.com)
  ⚬ Photosensor (TEMT6000, B07JB5TQ93, Comidox, $8.00 at Amazon.com for 4)
  ⚬ Multiconductor cable (24-14440, PRO POWER, $28.99 at Newark.com for 300 ft)
  ⚬ 5 min Epoxy (14250, Devcon, $10.35 at Grainger.com)
  ⚬ 15 min Black Plastic Bonder (50139, J-B Weld, $6.99 at Amazon.com)
  ⚬ (optional) Shrink tubing (HS101-1/16, Insultab, $3.03 at McMaster-Carr, Item 7496K82)
  ⚬ Connector receptacle 3 position (22-01-3037, MOLEX, $0.08 at Newark.com)
  ⚬ Connector receptacle 2 position (22-01-3027, MOLEX, $0.28 at Newark.com)
  ⚬ Connector headers 3 position (22-27-2031, MOLEX, $0.19 at Newark.com)
  ⚬ Connector headers 2 position (22-23-2021, MOLEX, $0.18 at Newark.com)
  ⚬ Contact sockets (08-50-0032, MOLEX, $0.18 at Newark.com)
  ⚬ 10 mL syringe (5100-X00V0, Henke-Ject, Henke Sass Wolf, Germany, $35.91 at VWR.com)
  ⚬ 1 mL syringe (309659, BD, $32.97 at VWR.com for 200)
  ⚬ Screw (3/16 in or 4.6 mm thread diameter, at least 5 cm long).
  ⚬ (optional) tubing (Tygon 3350 1/16 × 3/32, St. Gobain, $133 for 25 ft at Grainger.com)
  ⚬ (optional) matte tape (Magic Tape, Scotch™, $3.50 at Amazon.com)

### Materials and Equipment Used to for Data Acquisition

LabJack U3-HV ($169 at labjack.com)

## Methods

### Assembly of the ClampOD

**FIGURE 1:**
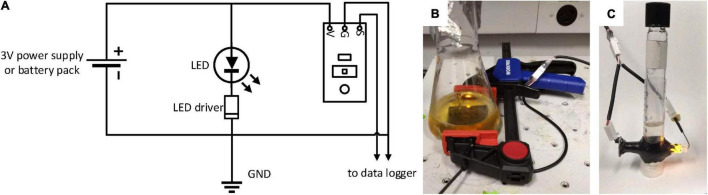
**(A)** Wiring scheme of the self-manufactured OD-meters, **(B)** photographs of a ClampOD attached to an Erlenmeyer shake flask, and **(C)** a TubeOD with a Hungate tube. GND: ground; V: voltage in; G: ground; S: signal output.

An amber light emitting diode (LED) (C503B-AAN-CY0B0251) with a typical dominant emission at 591 nm and a range of dominant wavelengths between 584 and 596 nm (for detailed spectral information see the manufacturers data sheet) was chosen as light source to match the commonly used 600 nm wavelength for cell density measurements (Figure 1, detailed and illustrated step-by-step procedure in Supplementary Materials). To ensure constant lighting of the LED independent of power fluctuations or decreasing battery levels, a LED driver (NSI45020AT1G) was soldered in series with the LED, resulting in a constant 20 mA current through the LED. A photosensor (TEMT6000) was used as detector. Light intensity was recorded as analog voltage between the ground and output pins of the photosensor. We used a multimeter for visual readings of light intensity and logged the data using a LabJack U3-HV (LabJack Corporation, Lakewood, CO, United States). For mobile application, a battery powered USB voltage logger could be used instead. LED and photosensor were powered in our case by a 3 V power supply (FBA_4330187065, SMAKNÂ), but can also be powered by 2 AA batteries. To exclude data variability introduced by movement and misalignment of light source and detector, the LED, photosensor, and sample need to be spatially fixed relative to each other for the duration of the experiment. To accommodate this requirement into a mobile device that can be used with a variety of culture vessels, light source and detector were mounted into holes drilled into a bar clamp (Workpro 6 inch barclamp W001411A). The 5 mm wide holes were drilled exactly opposite to each other to enable a direct light path between LED and photosensor. The LED was directly mounted into the hole and glued in place using 5-min epoxy. The photosensor was moved into place behind the opposite hole and a Viton O-ring was used between the sensor and the clamp to ensure a good fit and shield the sensor from ambient light. The positions of the O-ring and photosensor were adjusted, while LED and photosensor were switched on, to yield the highest light intensity and maximize the sensor sensitivity. The output voltage of the photosensor was measured with a multimeter and moved until the output was maximized. At this optimal position, the photosensor was glued in place with 5-min epoxy. After the glue was completely hardened, the cables of the photosensor and LED were connected to one 3-conductor cable that was used to power the device and record the data. Once the ClampOD is built, it can be attached to various culture vessels and record changes in light transmission (Figure 1B). If the culture vessel is thin and the sample clear, the light intensity reaching the detector can exceed the measurement range of the detector. In this case, one or two layers of matte tape can be attached to the clamping surface to reduce incident light intensity before starting measurements. The optimum light intensity is reached when the freshly inoculated culture (or blank medium) reduces the voltage output of the sensor to about 90% of the maximum output voltage.

### Assembly of the Tube-Photometer

LED and photosensor were assembled and connected as described above (detailed and illustrated step-by-step procedure in Supplementary Materials). The tube holder consisted of a cut-off 10 mL syringe (5100-X00V0, Henke-Ject, Henke sass wolf, Germany) fitted with a perpendicular cut-off 1 mL syringe (see Supplementary Materials). LED and photosensor were mounted on opposite sides of the 1 mL syringe tube. Because of the brightness of the LED and the short path length, the incident light intensity had to be reduced in many cases. This was achieved either by introducing one or two layers of matte Scotch™ tape between LED and tube, or by introducing a piece of tubing (Tygon 3350 1/16 × 3/32) of desired length into the 1 mL syringe piece between LED and Hungate tube. The assembled unit fit snugly around a standard Hungate tube (CLS-4208, Chemglass) (Figure 1C). Connection and data acquisition were performed as described above and shown in the SI.

### Online Data Acquisition

The output voltage of the photosensor depends on the input voltage (maximum output voltage = input voltage; 2–5 V) and decreases linearly with light intensity for more than two orders of magnitude according to manufacturer’s specification. The self-manufactured photometers were connected to a LabJack U3-HV (labjack.com) for data logging (see detailed description in Supplementary Materials). Data were recorded with the LJLogUD V1.20 (labjack.com) software and saved as CSV files for analysis with Microsoft Excel or MATLAB. For convenience, a jupyter notebook MATLAB template is available on GitHub^1^. Data acquisition frequency varied between 0.1 and 5 s to limit the total amount of data collected for each experiment. Alternatively, any data logger capable of recording voltages can be used to record the data, including mobile USB data loggers or serial port monitoring software. Point readings can be taken by attaching a multimeter. While not as convenient as online measurements, the culture conditions are not disturbed and the culture volume remains constant by clamping and manual multimeter reading for OD determination.

### Preparation of a Microbial Optical Density Calibration Solution

The ClampOD requires calibration against another standardized method for cell density determination for each online measurement series, to convert voltage output to cell densities. We prepared a calibration solution of the bacterium *Escherichia coli* [strain BL21(DE3)] by growing 2 L of cells in Luria-Bertani (LB) medium to an OD of 1.0, measured by a traditional benchtop photometer at 600 nm. Cells were then harvested *via* centrifugation, washed and resuspended in phosphate-buffered saline (PBS) and fixed with 2% paraformaldehyde overnight at 4°C. Cells were then washed twice in PBS and stored in PBS:ethanol (50:50 v/v) at 4°C until use. Cells were diluted in water to obtain solutions of different ODs. For the triplicate TubeOD calibration, the cells were diluted in 1X PBS.

### Data Analysis and Comparison of ClampOD and TubeOD Measurements to Commercial Photometer Readings

To assess the performance of the fabricated photometers, turbidity measurements taken with the ClampOD and TubeOD were compared to OD measurements performed by a commercial benchtop cuvette photometer at a wavelength of 600 nm (Ultrospec2100 pro, Amersham Biosciences, United Kingdom). For this comparison, each cultivation vessel was filled with different dilutions of the prepared fixed *E. coli* standards while kept inserted into a self-manufactured photometer. For each of these dilutions, the voltage output as well as the commercial photometer OD was recorded. To align with to the theoretical relationship of OD and light transmission (OD = log_10_ (1/T), voltage readings obtained by the self-manufactured OD-meters were log_10_ transformed and plotted against the commercial photometer ODs. To reduce variability in the voltage readings from scattering due to mixing or bubbles, the medians of every 25 data points were used to calculate OD for all growth curves.

### Reproducibility of Measurements

To evaluate the reproducibility of repeated measurements, the ClampOD was removed and replaced 5 times to a dilution series of fixed *E. coli* suspensions in either 15 mL Falcon tubes or 15 mL glass tubes. The voltage reading was recorded after each replacement. The mean voltage was calculated, and then each reading was subtracted from the mean to find the residuals. The long-term signal drift of the photosensor in ClampOD was assessed while a ClampOD was attached to an Erlenmeyer shake flask filled with water (blank measurement) and while being clamped onto a pen (any opaque object blocking light transmission completely can be used, dark measurement).

### Growth Curves

To obtain growth curves of different microorganisms in a variety of growth vessels, microbes were grown as described below.

*E. coli* [strain BL21(DE3)] was inoculated into 150 mL LB medium in a baffled 250 mL Erlenmeyer shake flask from a pre-culture stored at 4°C for several days. The culture was agitated at 100 rpm on an orbital shaker and grown at 30°C. To directly compare the growth curve recorded by the ClampOD to ODs determined in a commercial cuvette photometer, 1 mL samples were taken periodically, transferred into cuvettes and immediately measured without dilution as described below. After 6.5 h, 50 μg/mL tetracycline was added to inhibit growth before proceeding with the ClampOD calibration as described below. For calibration, the culture was diluted in LB medium containing 50 μg/mL tetracycline to inhibit growth of this fast growing microbe during the dilution procedure.

*Clostridium kluyveri* strain DSM555, an anaerobic bacterium converting acetate + ethanol to butyrate, was cultivated in a butyl rubber stopper sealed 120 mL serum vial with N_2_/CO_2_ (80/20 v/v) at atmospheric pressure and 50 mL of DSMZ medium 52, modified to include only 1.04 mM L-cysteine and 0.84 mM Na_2_S and pH adjusted with 1 mL 1 M NaHCO_3_. The culture was stirred with a magnetic stir bar at 120 rpm and incubated at 30°C. The ClampOD was attached 48 h after inoculation. For the calibration, the grown culture was diluted with 0.1X PBS.

*Methanococcus maripaludis* strain MM901, a strictly anaerobic hydrogenotrophic methanogenic archaeon (Costa et al., 2010), was grown in anaerobic 120 mL serum bottles containing 50 mL medium JD (Kracke et al., 2020) sealed with butyl rubber stoppers under a H_2_/CO_2_ (80/20 v/v) atmosphere pressurized to 10 psi. Additional H_2_/CO_2_ mix was added with a syringe after 24 h. The culture bottle was clamped with a ClampOD, mounted slightly tilted on an orbital shaker and agitated at 100 rpm at 30°C. For calibration, the grown culture was diluted stepwise with fresh anoxic medium using a syringe.

*Thermoanaerobacter kivui* TKV002, a strictly anaerobic thermophilic homoacetogenic bacterium (Basen et al., 2018), was grown in medium containing: 25.0 mM MES, free acid, 75.0 mM MES, sodium salt, 13.7 mM NaCl, 0.8 mM MgSO_4_ × 7 H_2_O, 18.7 mM NH_4_Cl, 1.3 mM KCl, 0.1 mM CaCl_2_ × 2 H_2_O, 0.7 mM KH_2_PO_4_, 40 μM uracil, 1 mL/L trace element solution SL10, 1 mL/L selenate-tungstate solution, 1 mM Na_2_S, 0.5 mg/L resazurin, and 20 mM glucose as carbon and energy source. Hungate tubes containing 3 mL medium and a small magnetic stir bar were inoculated with 300 μL actively growing culture and incubated at 65°C on a magnetic stir plate to prevent biomass settling. Growth curves recorded with the TubeOD within the 65°C incubator and connected with shielded cables to the LabJack U3-HV for data logging outside the incubator (see Supplementary Materials). For calibration, the grown cultures were diluted stepwise with fresh anoxic medium using a syringe.

*Lactococcus lactis* spp. *cremoris* wild type (NZ9000), a microaerophilic lactic acid bacterium, and the derived mutant Δ*ldhA* (NZ9020) (Bachmann et al., 2013) were cultivated in 10 mL of chemically defined medium (CDM) (Goel et al., 2012) supplemented with 1.5% casamino acids (w/v), 26 mg/L L-tryptophan, and 50 mM glucose for liquid cultures. Growth curves were started with a 1% inoculum from a starter culture in stationary phase for 24 h. Starter cultures of NZ9000 or NZ9020 were inoculated from a single colony from Difco M17 broth with addition of 25 mM glucose (GM17) or GM17 and 5 ug/mL erythromycin plates, respectively. All liquid cultures were grown statically at 30°C in Hungate tubes closed with screwcaps to facilitate microaerobic conditions. Growth curves were recorded with TubeODs. For calibration, the grown cultures were diluted aerobically with PBS buffer.

**FIGURE 2:**
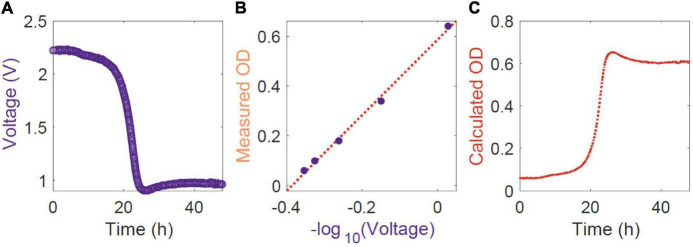
Work flow of data analysis. **(A)** The raw voltage output of the self-fabricated OD-meters TubeOD or ClampOD was logged over time. **(B)** A calibration curve of log_10_ transformed OD-meter voltage output to OD measurements in the commercial spectrophotometer at a wavelength of 600 nm was constructed (filled circles) and a linear (dotted line) or 2nd order polynomial regression function (not applicable here) was determined. **(C)** The corresponding calculated cuvette photometer OD was determined from the OD-meter output voltage and plotted as classical growth curve.

After obtaining the voltage data over time (Figure 2A), cells were diluted stepwise with medium or appropriate buffers in the same culture vessels while the ClampOD was still attached, or the tube still in the TubeOD assembly. Voltage readings of each dilution were recorded and plotted against absorption measurements obtained using a commercial photometer (Ultrospec2100 pro, Amersham Biosciences, United Kingdom) (Figure 2B). The latter samples were measured in 1.5 mL semi-micro cuvettes of 1 cm path length (Fisherbrand 14955127, Fisher Scientific, United States) at a wavelength of 600 nm. This quick calibration procedure eliminates bias based on different clamping between measurements or variations between culture vessels and allows the generation of growth curves (Figure 2C).

Voltage and Ultraspec2100 OD data were analyzed in MATLAB (ver. R2021a) using the fit function with 1st and 2nd order fits. Residuals were checked visually for normality with the histfit function and the root mean squared error was calculated for each calibration. A link to the MATLAB code is included in Supplementary Materials.

## Results

### Comparison of ClampOD and TubeOD Measurements to Optical Density Determination of a Commercial Cuvette Spectrophotometer

**FIGURE 3:**
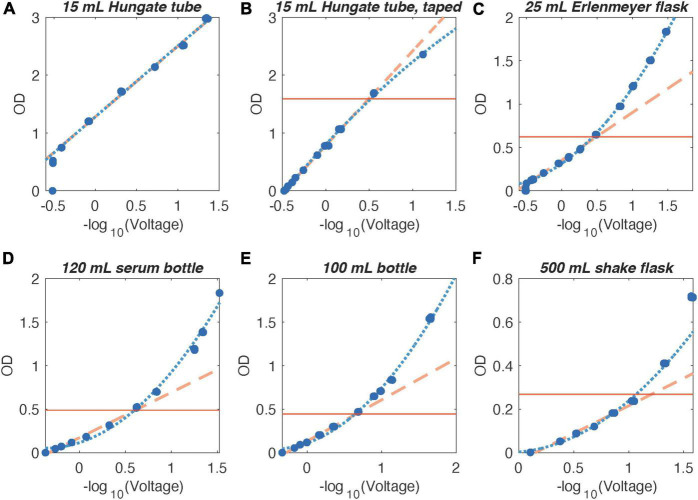
Correlation of OD values measured by the commercial cuvette spectrophotometer at a wavelength of 600 nm and log_10_ transformed ClampOD output voltage for fixed *E. coli* cell suspensions in various growth vessels. Dashed yellow lines: extrapolated linear fits to the linear portion of the correlation where ClampOD is equivalently accurate as the bench-top photometer; blue dotted lines: second order polynomial fit to data points within the extended detection range. The horizontal red lines indicate the OD⋅length = 2.5 threshold indicating the accuracy limit of the OD meters and coincides well with the OD at which the two fits diverge significantly. **(A)** Hungate tube, path length = 1.57 cm, **(B)** Hungate tube with tape, path length = 1.57 cm, **(C)** 25 mL Erlenmeyer flask, path length = 4.02 cm, **(D)** 120 ml serum vial, path length = 5.13 cm, **(E)** 100 mL bottle, path length = 5.62 cm, and **(F)** 500 mL baffled shake flask, path length = 9.33 cm.

Turbidity measurements of a dilution series of fixed *E. coli* cells showed a linear relationship of ClampOD and TubeOD output [-log_10_(voltage)] to OD values determined by the commercial cuvette spectrophotometer Ultrospec2100 pro for short path lengths (e.g., Hungate tubes). However, when the incident light intensity is too high, the photosensor is saturated and signal intensity is not increasing proportionally to cell density (Figure 3A). When the incident light intensity was reduced to a blank reading of about 90% of the maximum, ClampOD and TubeOD output correlated perfectly with cuvette-based OD measurements over a range from *OD* = 0 to about *OD* = 2 (Figure 3B). This indicates that for comparable path lengths, the self-made photometers produce results with similar accuracy as the commercial spectrophotometer. For larger culture vessels with longer light path length, this linear relationship applies only to lower ODs and the relationship becomes non-linear for higher ODs (Figures 3C–F). This deviation from linearity started at lower ODs for longer path lengths (Figures 3C–F).

### Detection Limits

The lower detection limit (blank) depended on the path length. A low path length resulted in oversaturation of the detector signal up to ODs of 0.4 (Falcon tube, data not shown) or 0.7 (Hungate tube) (Figure 3A). In these cases, decreasing the incident light intensity until the voltage output of the photosensor approached about 90% of the maximum value allowed accurate recording of lower ODs and resulted in a linear correlation of ODs with cuvette-based measurements over the entire measurement range (Figure 3B). This was achieved by applying one to three layers of opaque tape (ClampOD) or by fitting a piece of tubing into the 1 mL syringe part on the LED side (TubeOD, see Supplementary Materials).

The upper detection limit (maximum OD) was comparable to the commercial cuvette-based photometer detection limit for small path lengths. In Hungate tubes, OD > 2 was still detectable as accurately as with the commercial photometer (Figures 3A,B). Longer path lengths caused by larger diameter culture vessels decreased the maximum OD that could be determined reliably using the ClampOD (Figures 3C–E), in accordance with the Beer-Lambert law. In general, our data indicated that when the photosensor voltage output drops to about less than 0.3% of the lower detection limit [corresponding to OD * length (cm) > 2.5], the signal to noise ratio became too large for accurate data interpretation. However, even above this accuracy limit, OD changes could be inferred, albeit at lower accuracy with the help of a second order polynomial fit (extended detection range).

### Reproducibility of Measurements

Reproducibility of measurements with the ClampOD was determined by repeated clamping and removal of the device on a Hungate tube with fixed *E. coli* suspensions and recording voltage values. The mean residual was 0.003 V with a standard deviation of 0.0141 and the max residual was 0.024 V (see Supplementary Figure 1). Compared with the voltage output range of the detector (3 V with a 3 V power supply and a measurable range of 2.4 V on a low voltage channel of the LabJack), repeated clamping will introduce errors of less than 1% of the measurement range. However, re-clamping can cause breaks in growth curves and is not recommended for online growth curve measurements (data not shown).

### Other Parameters to Consider

The photometers were temperature sensitive, and the output voltage changed when transferred to different temperatures. Therefore, acclimating the photometer at a given temperature before starting measurements is necessary to avoid temperature-induced signal drift. This is also commonly observed for commercial benchtop photometers.

Long-term operation measuring a blank sample resulted in an average signal of 1.14 V and drifted by less than 0.2 mV per hour and signal drift under dark current conditions (i.e., clamped to a pen) was not detectable.

Due to the continuous illumination of the sample in this kind of photometers, it could be unsuitable for measurement of light sensitive organisms. In addition, constant illumination might affect experiments with phototrophic organisms that can utilize the wavelength emitted by the LED and lead to additional growth and light absorption by pigments.

### Growth Curves

**FIGURE 4:**
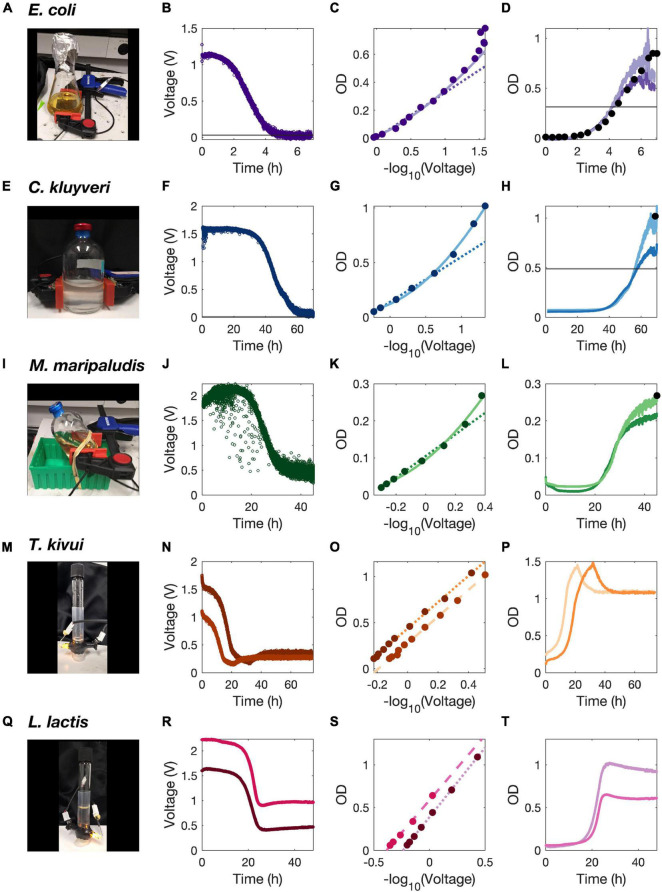
Growth curve measurements of different microbial cultures. Growth of *E. coli* **(A–D)**, *C. kluyveri* **(E–H)**, and *M. maripaludis* **(I–L)** was measured with the ClampOD. Growth of *T. kivui* **(M–P)** and *L. lactis* wild type (darker purple colors) and ΔldhA mutant (pink) **(Q–T)** was measured with the TubeOD. Photos of each experimental setup are shown **(A,E,I,M,Q)**. Raw voltage output from the photosensor was recorded over time (open circles) **(B,F,J,N,R)**. After recording the growth curves, the cultures were diluted and the OD-meter output was calibrated against a commercial cuvette photometer at a wavelength of 600 nm (filled circles). These points were fitted with first (dotted and dashed lines) and/or second order polynomial regression lines (solid lines) **(C,G,K,O,S)**. Based on these regressions, growth curves were plotted using calculated (cuvette photometer equivalent) ODs **(D,H,L,P,T)**. Black filled circles indicate ODs determined simultaneously in a commercial cuvette photometer **(D,H,L)**. Lighter colors represent growth curves calculated from the second order polynomial fit, darker shades growth curves calculated from the linear polynomial fit **(D,H,L)**. The growth curve color corresponds to the strain or replicate calibration fit **(P,T)**.

We recorded growth of various microbes continuously in different growth vessels using the ClampOD and TubeOD photometers. The ClampOD was used to record growth of *E. coli* agitated on a shaker in a 150 mL Erlenmeyer shake flask (Figures 4A–D) and data were compared to simultaneously obtained manual OD measurements. The bacterium *Clostridium kluyveri* was monitored with the ClampOD growing on acetate + ethanol in a stirred anaerobic serum bottle (Figures 4E–H), as well as the hydrogenothrophic methanogen *Methanococcus maripaludis* growing on H_2_ + CO_2_ in a shaking serum bottle (Figures 4I–L). The TubeOD was used to record growth curves of *Thermoanaerobaer kivui* at 65°C (Figures 4M–P) in an incubator and of *Lactococcus lactis* at 30°C (Figures 4Q–T).

After growth of the cultures, they were serially diluted and measured for comparison simultaneously in the OD-meter and commercial photometer, yielding calibration curves that were used to convert the output voltage into equivalent cuvette-photometer ODs. For the TubeOD, an excellent linear, correlation was observed between the logarithm of the output voltage and OD values measured in a commercial photometer (Figures 4O,S), which is in good agreement with the fixed *E. coli* calibrations (Figures 3A,B). For the ClampOD attached to larger diameter growth vessels, such as serum vials or shake flasks, only the highest dilutions fell into the linear range. Therefore, a polynomial fit of second order was used to estimate ODs over an extended range (Figures 4C,G,K).

A growth curve of *E. coli* growing in a 250 mL baffled shake flask (8 cm diameter) recorded with the ClampOD (Figure 4A) further demonstrated the limitations of accurately determining cell densities in large diameter culture vessels with the ClampOD. Already at ODs of less than 0.6, the ClampOD reached its upper detection limit and the signal to noise ratio becomes too low for accurate OD determination. The first few dilution steps for the calibration did not result in a measurable decrease in signal intensity further corroborating that the upper detection limit was reached. Manually measured cuvette-based ODs of samples taking at several time points during growth show that the ClampOD values start to significantly deviate from the manual measurements at OD values around 0.25 (Figure 4D). This further underlines the limitations of accurately determining higher ODs at larger path lengths (= large culture vessels). Nevertheless, this equipment is suitable to obtain accurate information about growth rate from the data obtained at low OD. Growth rates obtained by the linear fit and the polynomial fit were similar (1.71 h^–1^, 1.77 h^–1^).

Growth of the strictly anaerobic, chain elongating *C. kluyveri* was monitored with a ClampOD attached to a 120 mL serum bottle on a magnetic stir plate to avoid settling of the slow growing strain while recording the growth curve (Figure 4E). After a lag phase of 30 h, the strain grew slowly over more than 24 h to an OD of about 1. Again, a 2nd order polynomial fit was used to calculate OD values (Figure 4G). Light attenuation due to microbial light scatter approached the detection limit of the photosensor, which approached an output of 0 V (Figure 4F). In addition, the measurement error and noise increased significantly at OD values above 0.75, indicating that despite the acceptable calibration curve, calculated OD values above 0.75 need to be treated with caution and that these turbidities approach the physical limitations of light scatter-based biomass measurements and the upper detection limit of the ClampOD. However, growth rates calculated from the linear and polynomial fit were very similar with 0.127 h^–1^ and 0.126 h^–1^, respectively.

*M. maripaludis* growth on a H_2_/CO_2_ gas mix in a 120 mL serum bottle on a shaker was recorded with a ClampOD (Figure 4I). The raw data show outliers that were caused by gas bubbles or gas mixed into the solution by shaking (Figure 4J). After an initial drop in OD and a lag phase of about 20 h, the strain grew to a final OD of about 0.25. Even this low OD was calculated using a 2nd order polynomial fit, due to the long path length of 5 cm. However, the entire growth curve was within the detection range of the photosensor and the maximum OD obtained with the polynomial fit is similar to the cuvette-based OD measured at the end of the growth curve (Figures 4J–L). Again, growth rates calculated from the linear and polynomial fit were very similar with 0.268 and 0.270 h^–1^, respectively.

Growth of *T. kivui* cultures under strictly anaerobic conditions was compared at 65°C using parallel TubeODs and a linear calibration. After a lag phase of 2 and 5 h, the cultures grew for 19 and 26 h, before the OD dropped sharply to stabilize in late exponential phase. The maximum growth yields (OD = 1.5) as well as the growth rates (0.251 and 0.248 h^–1^) were very similar between the biological replicates in different TubeODs, demonstrating comparability between data obtained by different TubeODs (Figures 4M–P).

The obtained growth curves of *L. lactis* using the TubeOD (Figure 4Q) and linear calibrations clearly showed a lag time of more than 15 h before either strains started growing for another 15 h and then decreasing in OD before stabilizing in late stationary phase (Figures 4R–T). The growth rate of the Δ*ldhA* mutant was calculated to be slightly higher with 0.372 h^–1^ compared to 0.363 h^–1^ for the wild type. While this growth rate difference is less pronounced than published elsewhere (Bachmann et al., 2013), we observed similar growth rates for wild type and Δ*ldhA* mutant in several independent experiments not using our self-assembles photometers (data not shown). The maximum OD of both strains differed significantly, with the Δ*ldhA* mutant achieving higher growth yields (Figures 4R–T). These differences in growth yields correspond well to results obtained elsewhere (Bachmann et al., 2013).

Taken together, these growth curves showed that TubeOD and ClampOD record high quality growth curves with exceptional time resolution. Both low-cost photometers accurately monitored the growth of diverse microbes with different physiologies, growth rates, and growth yields in various cultivation vessels—at wide ranges of ODs in low diameters and at low ODs in vessels with larger diameters.

## Discussion

We developed two simple photometers for online measurements of microbial cell densities. The TubeOD photometer was developed out of the necessity to record replicate growth curves of strictly anaerobic and potentially thermophilic cultures in Hungate tubes. While there are some commercial products available that offer similar functionality (Sasidharan et al., 2018), the cost of TubeOD is substantially lower, even if all the assembly tools need to be purchased and including parts only available in bulk. In addition, the simple design can be easily modified to fit individual needs. In our hands, assembly of three TubeOD photometers took less than 1 day, including glue drying times and total cost of each photometer was less than $10 for parts. The cheap and fast manufacturing, if combined with the LabJack U3 for online data acquisition, allows data collection from 16 TubeOD photometers simultaneously. This allows replicate, high resolution, side-by-side comparisons of the effect of substrates, media composition, temperature, or other environmental factors on the growth of a variety of microbes, including strictly anaerobic microbes and thermophiles without any hands-on time for data acquisition. This gains even more impact on productivity considering the effort required to monitor slow growing anaerobes with variable lag times that complicates sampling the desired growth phase and to acquire dense high-quality data to describe their growth. In addition, online measurement prevents perturbation of systems that are sensitive toward invasive sampling methods.

While the ClampOD provided high quality data with short path lengths over a wide OD range (i.e., in linear calibration range), its main feature is the flexibility of measuring larger diameter growth vessels. However, the maximum detectable OD decreases with increasing light path length and data become less accurate (i.e., in non-linear calibration range). On the other hand, in all our experiments, accurate growth rates could be obtained from the growth curves, because the exponential phase started at low OD (always in the linear calibration range) and was accurately captured by our OD-meters. Nevertheless, even in the non-linear range the ClampOD proved invaluable for many practical applications, including monitoring cell density in different chemostat systems, determining late exponential phase of serum bottle cultures, or monitoring pre-cultures to evaluate growth stage or time in stationary phase of pre-cultures after overnight and weekend growth. In general, the main benefit of the ClampOD is the flexibility that allows estimation of microbial cell density in virtually any growth vessel of interest.

The flexibility to attach the ClampOD to various culture vessels inherently changes the light path and the calibration for each measurement. Therefore, we strongly recommend calibrating the output of each ClampOD under the exact same conditions that have been used during the growth curve measurement to either a standard benchtop photometer, or any other standardized parameter describing cell density (e.g., protein concentration, total organic carbon, cell dry mass). This can be easily achieved by diluting the grown culture in small steps with an appropriate medium or buffer, without removing the sample from the ClampOD. Such calibration ensures accurate conversion of the measured light extinction to desired cell density specific for the strain and conditions investigated over the entire growth curve, if within the detection range of the ClampOD. This calibration also identifies the upper detection limit for each cultivation vessel, if the culture exceeded the maximum detectable OD. However, even this calibration might have to be treated with caution when the investigated cells considerably change their shape or surface structure (Stevenson et al., 2016). The same calibration should be performed for the TubeOD to ensure accuracy of the data.

It is important to note that some OD values reported here cannot be used directly to estimate cell density. Online-measured OD values often exceed the range (0–0.3) of linear correlation between OD and cell density (Koch, 2007). Consequently, an additional non-linear calibration curve of cuvette photometer OD to cell density is needed, or all samples have to be diluted to fall into the linear range and the true OD can then be calculated from the dilution factor (Koch, 2007). With this additional calibration step, the obtained TubeOD and ClampOD values could be translated into true cell density values. However, the purpose of this study was to describe and test simple and cost-effective “do-it-yourself” photometers, which can best be accomplished by comparison to a commercial product.

Due to the inherent decrease in accuracy of OD measurements with increasing cell density, online growth measurements are bound to yield unreliable results when the OD exceeds the measurement range. The only viable way for accurate online OD measurements at higher cell densities would be to decrease the path length, which would require the construction of sophisticated growth vessels, expensive sensors, or flow through measuring cells (Junker et al., 1994; Koch, 2007). Alternatively, other methods can be used to determine cell densities, such as backscatter measurements, nephelometry, and capacitance-based methods (Junker et al., 1994). Further, changes in medium composition based on microbial activity can be monitored including conductivity, pH, or color (Mauerhofer et al., 2019). However, these methods also require sophisticated equipment or sensors, are not easy or cheap to implemented in small-scale lab settings, and are usually limited to certain microbes, metabolic pathways, or medium (Mauerhofer et al., 2019).

The here presented photometers have further benefits compared to others: (1) our photometer designs do not require 3D printing or prototyping the sample holder, which is not commonly available in biological labs, (2) the output of the photosensor can be recorded with a variety of devices, and no programming skills are needed to analyze the data, (3) within inherent physical limits, the ClampOD can be used on any transparent culture vessel. In general, the ease of manufacturing these OD-meters, combined with the low cost and versatility, should enable many microbiologists and students to record continuous high quality growth data while saving time compared to the standard technique using a benchtop cuvette photometer.

## Data Availability Statement

The raw data supporting the conclusions of this article will be made available by the authors, without undue reservation.

## Author Contributions

JD conceived, designed, built the photometers, collected, analyzed data, and wrote the publication. GC performed calibrations and growth curves, analyzed data, and wrote the manuscript. WG built the photometers and obtained growth curves. AMü, AMc, and FK obtained growth curves, provided strains and media, and improved the photometers. JA supplied *C. kluyveri* and DSMZ medium 52. All authors read, edited, and approved the final manuscript.

## Conflict of Interest

The authors declare that the research was conducted in the absence of any commercial or financial relationships that could be construed as a potential conflict of interest.

## Publisher’s Note

All claims expressed in this article are solely those of the authors and do not necessarily represent those of their affiliated organizations, or those of the publisher, the editors and the reviewers. Any product that may be evaluated in this article, or claim that may be made by its manufacturer, is not guaranteed or endorsed by the publisher.

## Footnotes

1
https://github.com/gcallander/OnlineOD
