# Supplementary material for: Low-Cost Clamp-On Photometers (ClampOD) and Tube Photometers (TubeOD) for Online Cell Density Determination
Source: Front Microbiol. 2022 Jan 13;12:790576. doi: 10.3389/fmicb.2021.790576 (PMC8793360; doi:10.3389/fmicb.2021.790576)
Supplement: Supplementary file 1 [file Table_1.DOCX]

Supplementary Information to

# Low-cost clamp-on photometers (ClampOD) and tube photometers (TubeOD) for online cell density determination

Jörg S. Deutzmann^1*^, Grace Callander^2^, Wenyu Gu^1^, Albert L. Müller^1^, Alexandra L. McCully^1^, Jenna Kim Ahn^2^, Frauke Kracke^1^, Alfred M. Spormann ^1,2*^

^1^ Civil and Environmental Engineering, Stanford University, Stanford, CA, USA

^2^ Chemical Engineering, Stanford University, Stanford, CA, USA

## Supplementary Figure 1


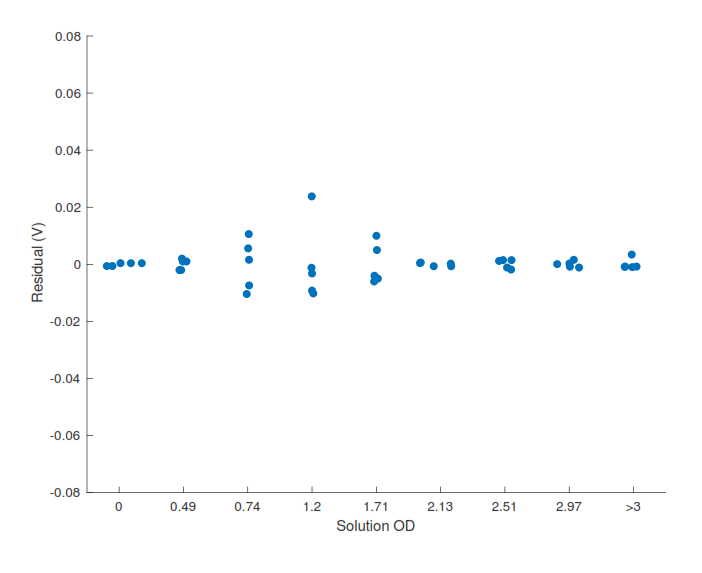


*Supplementary Figure 1. Reproducibility of measurements is illustrated as residuals for 5 repeated measurements of E. coli suspensions of different cell density. Repeated clamping and removal of the ClampOD resulted in reproducible OD reading across the measurable OD range.*

## Step by step fabrication of clamp-on photometer

Equipment used/needed:

- Soldering station or conductive glue (e.g. silver epoxy)
- Pliers
- Wire stripper (or scissors)
- Screwdriver
- Parts
  - LED (C503B-AAN-CY0B0251, CREE LED, $0.15 at at Newark.com)
  - LED driver (NSI45020AT1G, ON SEMICONDUCTOR, $0.52 at Newark.com)
  - Photosensor (TEMT6000, B07JB5TQ93, Comidox, $8.00 at Amazon.com for 4)
  - Multiconductor cable (24-14440, PRO POWER, $28.99 at Newark.com for 300 ft)
  - 5 min Epoxy (14250, Devcon, $10.35 at Grainger.com)
  - (optional) Shrink tubing (HS101-1/16, Insultab, $3.03 at McMaster-Carr, Item 7496K82)
  - Connector receptacle 3 position (22-01-3037, MOLEX, $0.08 at Newark.com)
  - Connector headers 3 position (22-27-2031, MOLEX, $0.19 at Newark.com)
  - Contact sockets (08-50-0032, MOLEX, $0.18 at Newark.com)
  - Clamp (‎W001411A, HANZGHOU GREATSTAR INDUSTRIAL CO.LTD, $39.99 at Amazon.com for 6)
  - O-ring (0.070” dash number 009, $6.63 at McMaster-Carr, Item 9464K14)

The shorter (-) leg of the LED was soldered to the LED driver with the marked side (cathode, -) facing away from the LED. If no soldering station is available, gluing with conductive silver epoxy is an acceptable alternative. The LED driver delivers a constant current through the LED, which allows constant brightness of the LED even when powered with different voltages (between ca. 2.5 and 45 V) and prevents dimming or burnout of the LED.


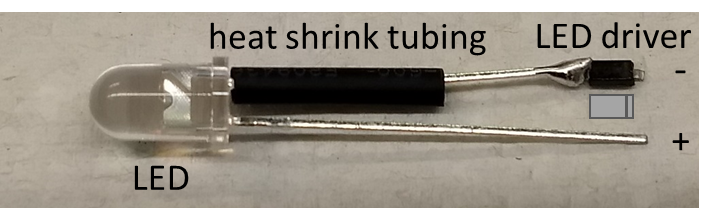


*Supplementary Figure 2: LED with LED driver soldered onto the shorter (-) leg. The LED driver was soldered on with the marking (line) facing away from the LED. Heat shrink tubing was put on the shorter leg before soldering the LED driver (to later cover and support the connection and isolate again short circuiting)*

The LED + LED driver assembly was soldered to a 2-conductor cable (ours was scavenged from an old 12 V DC power supply), and all soldered connections were covered in glue (gorilla glue or epoxy) and further covered with heat shrink-wrap and heated. Gluing and covering the connections is optional but adds rigidity to the connection and prevents the soldered connections from loosening easily. If the cable does not have marking to distinguish the sides (here a white line) make sure to label which sides are + and -, especially if the two legs might look identical after applying the cover layer of glue and heat shrink tubing.


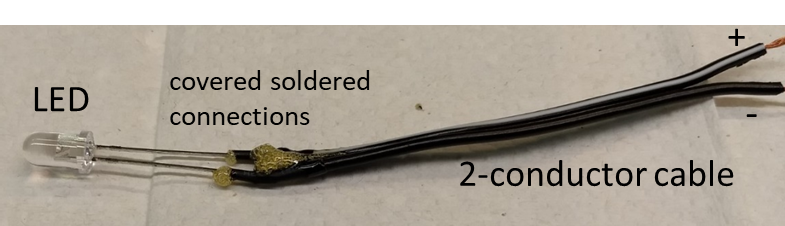


*Supplementary Figure 3. The LED + LED driver assembly was soldered to a 2 conductor cable. Note the covered soldered connections.*

The connections of the photosensor TEMT6000 were soldered directly to a 3-conductor shielded cable (Propower microphone cable 24-14440, Newark.com, Chicago, IL, USA). The connections were soldered to the back of the detector. We connected white to the sensor output, red to VCC (+), and bare wire to ground (-).


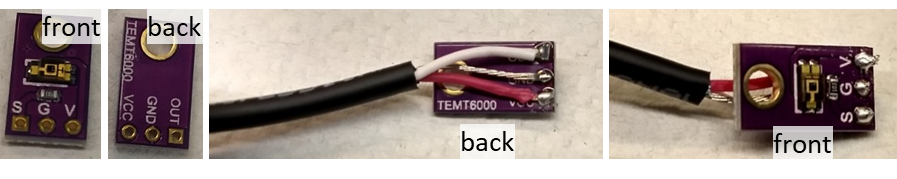


*Supplementary Figure 4. The TEMT6000 photosensor was soldered to a 3 conductor cable on the back side by removing a few mm of insulation from each conductor, sticking the bare wire ends through the holes of the TEMT6000 and soldering the wires in place.*

To create a straight light path, holes were drilled on opposite sides of the clamping surfaces of the wood clamp (Workpro 6 inch barclamp W001411A) such that they face each other and align well when the clamp is completely closed. To this end, one hole was drilled through the clamping surface of the opened clamp. The clamp was closed and the opposite side was marked with a pen through the first drilled hole. The second hole was drilled on the marked spot. Subsequently, the red gripper pads were similarly marked and drilled through to create one long tunnel through the closed clamp


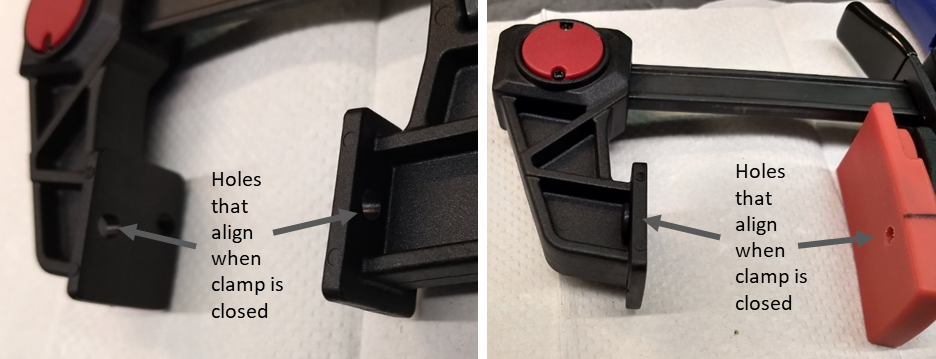


*Supplementary Figure 5. Holes were drilled through the clamping surfaces of the wood clamps and through the gripper pads.*

Next, the LED assembly was inserted into one hole (here the hole on the side closer to the handle), connected to a power supply (long leg to +, short leg with LED driver to -) and adjusted to shine light centered to around the opposite hole. The LED needed to be inserted only into the hard plastic part of the clamp and cannot protrude trough the rubber grip pad, lest the LED prevent effective clamping or get damaged by the clamping pressure.


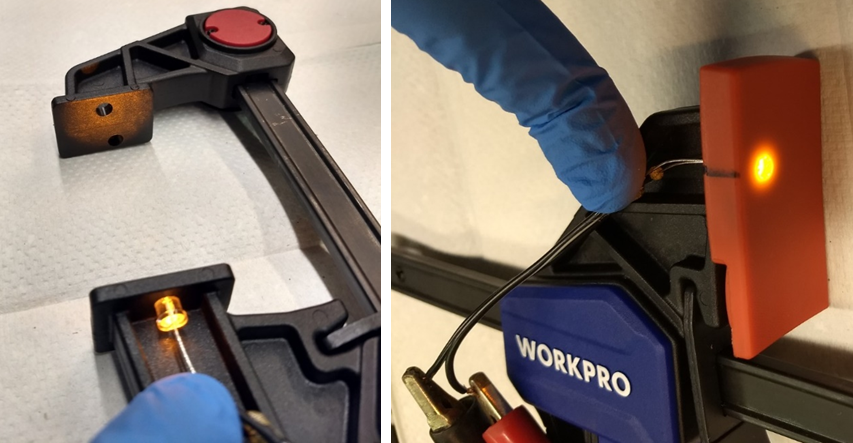


*Supplementary Figure 6. The LED was inserted into the hole in one of the clamping surfaces of the wood clamp. Care was taken that the LED did not protrude through the red gripper pad.*

Fix the LED in place with 5 min epoxy, letting the Epoxy harden while holding the LED light cone centered on the opposite hole.


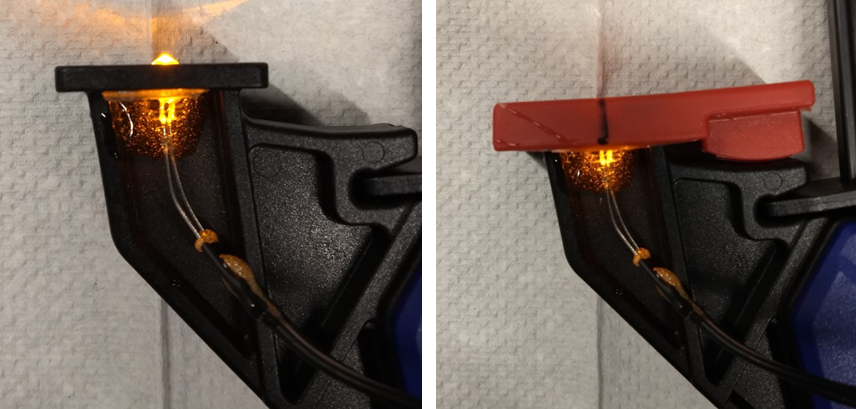


*Supplementary Figure 7. The Led assemble was glued into place using 5 min epoxy. The light cone of the LED was held centered around the hole in the opposite clamping pad while the epoxy hardened to make sure most of the LED light reached the detector. The LED protruded a bit through the hole, but the gripping pad completely covered the protruding part and still allowed efficient clamping of culture vials.*

Next, the photosensor was mounted behind the hole on the other side of the clamp. A spacer (Viton O-ring, 9 mm diameter, 1.8 mm thick) sealed the sensor against ambient light and residual epoxy resin and allowed to mount the sensor parallel to the clamp surface despite the protruding soldering points on the front side. The light sensor of the TEMT6000 should be mounted central behind the hole.


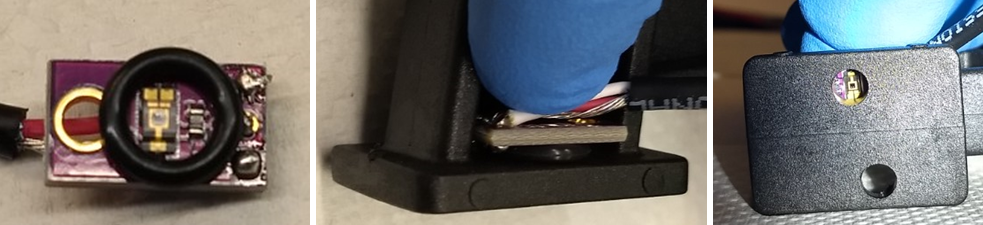


*Supplementary Figure 8. The photosensor was mounted centered behind the other hole in the clamp (right panel, the dark square dot of the light sensor is visible through the hole; please disregard the second hole. It was drilled by accident). An o-ring was inserted between sensor and clamp (left and middle panel) to block ambient light from the sides and to provide a cushion so that the sensor can be glued in place parallel to the clamping surface and is not angled by the protruding soldering sites.*

The sensor was glued in place using 5-min epoxy. The glue hardened while the sensor was held in place with a toothpick and by holding the connecting cable in place.


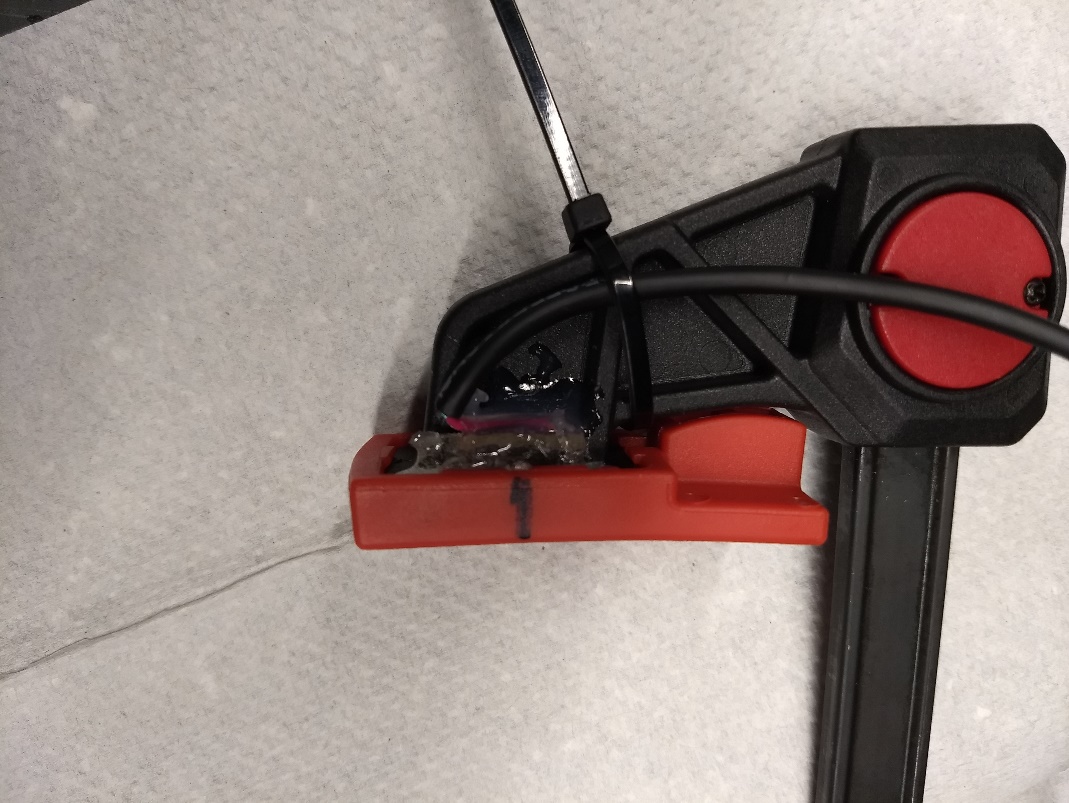


*Supplementary Figure 9. The Photosensor was glued in place and the connecting cable held in place with a zip tie for convenience.*

To complete the wiring, it was practical to combine the wires from LED and sensor into a 3-conductor plug. To this end, we twisted the bare wire from the photosensor (ground) together with the negative wire from the LED and fixed the twisted ends with crimp type contact sockets (MOLEX 08-50-0032). The red wire from the sensor (VCC, positive) was twisted together with the positive wire from the LED and crimped the same way. The white wire from the sensor (connected to “out”) was also crimped. We soldered all connections to the contact sockets to secure them in place. These contact sockets were then inserted into their plastic housing (MOLEX 22-01-3037). This created a plug that allows connecting this clamp-on photometer easily to a power supply and data logging device.


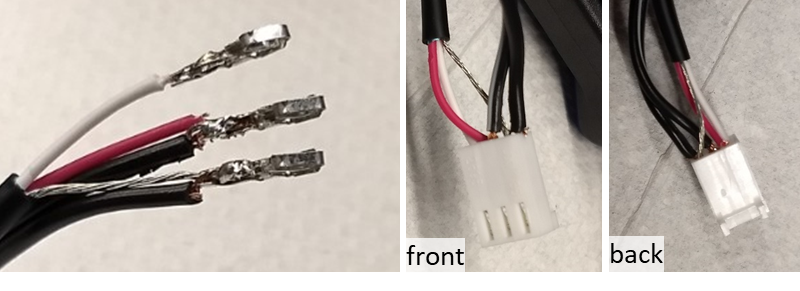


*Supplementary Figure 10. The conductor wires leading to LED and sensor were combined and inserted into a molex 3 position plug. The wire connected to “out” or “S” on the detector (white wire, left panel upper wire) was capped with a crimp type contact socket. The red wire from the sensor (VCC, positive) was twisted together with the positive wire (white stripe) from the LED and capped (left panel, middle wires). The bare wire from the photosensor (ground) was twisted together with the negative wire from the LED and capped (left panel, bottom wires). The crimp caps were inserted into the connector plug housing (middle and right panels)*

To connect the clamp photometer to power and data logging, we manufactured extension cords of different lengths, depending on the application. The extension cords consisted of a 3-conductor cable with the 3 conductors soldered to a 3-position connector header and free wires on the other side to connect to the screw terminals on data logger and power supply adapter.


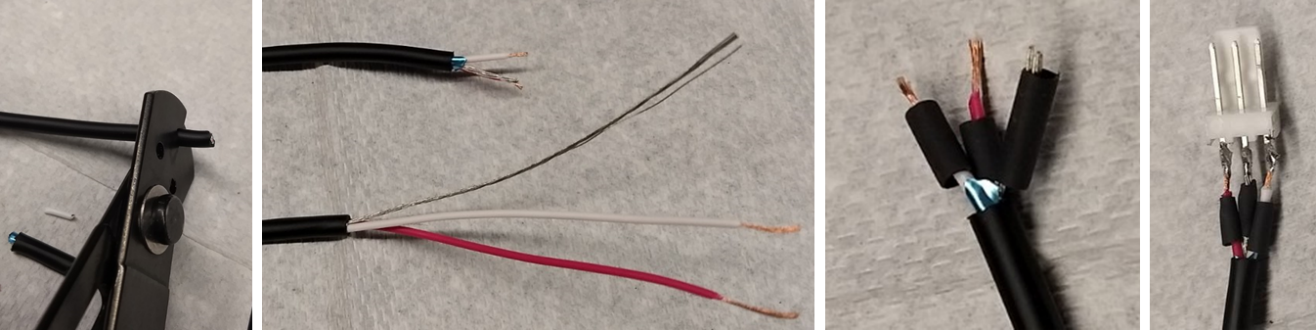


*Supplementary Figure 11. construction of the connection cable. 1.5 cm of the outer insulation was removed on one side and 10 cm on the other side of the 3 conductor cable and 0.5-1 cm of the insulation of the two insulated inner conductors (red and white) was removed (left and second panel). Heat shrink tubing was threaded on the short ends to later insulate the soldered connections against each other (third panel). The shorter ends were then soldered to a 3 position connector header and the connections insulated against each other using glue and heat shrink tubing. Matching the wire colors to the colors used in the plug on the clamp photometer is beneficial to avoid confusion.*

We used a standard 3 V power supply (FBA_4330187065, SMAKNÂ) with a screw terminal-2.1x5.5MM DC Power Jack adapter (B01J1WZENK, DAYKIT through Amazon.com) and a LabJack U3-HV as power source and data logger, respectively. The negative lead from the power supply was connected to ground of the LabJack and to ground/negative of photosensor and LED. The positive lead from the power supply was connected to VCC/positive of sensor and LED and the output from the sensor was connected to an analog input channel of the LabJack.


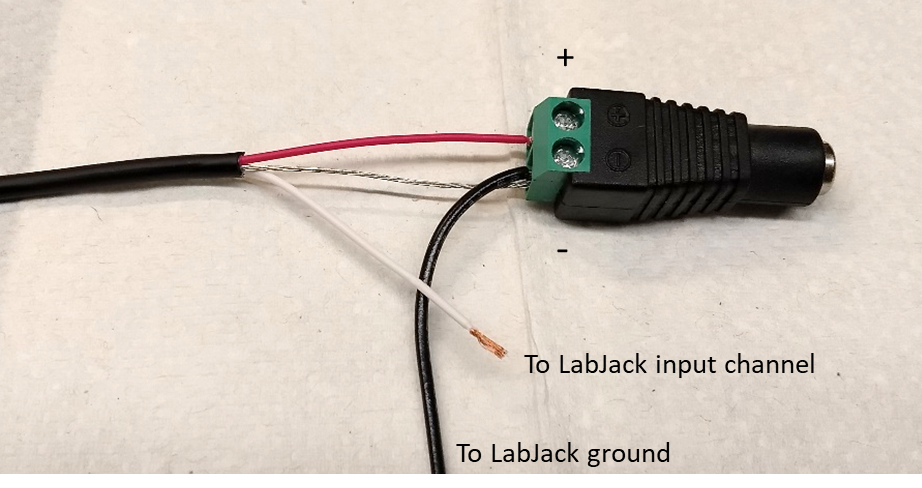


*Supplementary Figure 12. Wiring of the connection cable to the power supply and data logger. The conductor wire connecting to VCC/positive of sensor and LED was connected to the + terminal on the power plug adapter. The conductor wire connecting to the output (“out” or “S) on the sensor was connected to a input channel on the LabJack. The conductor wire connecting to “–“ on the LED and to ground G on the sensor was connected to the negative – terminal of the power plug adapter an with an additional wire to the ground terminal at the LabJack.*

It is important to note that the low voltage channels of the LabJack only record voltage between 0 and 2.4 V. Therefore, the maximum voltage recorded by the sensor should be lower than 2.4 V. When needed, this was achieved by attenuating the LED with opaque tape or tubing until the sensor reading of a water or blank medium filled culture vessel was below 2.4 V (see above).

## Step by step fabrication of the TubeOD photometer

Equipment needed”

- Soldering station or conductive glue (e.g. silver epoxy)
- Pliers
- Wire stripper (or scissors)
- Razor blade
- Cork borer (Humbold 3276G40)
- Parts
  - LED (C503B-AAN-CY0B0251, CREE LED, $0.15 at at Newark.com)
  - LED driver (NSI45020AT1G, ON SEMICONDUCTOR, $0.52 at Newark.com)
  - Photosensor (TEMT6000, B07JB5TQ93, Comidox, $8.00 at Amazon.com for 4)
  - Multiconductor cable (24-14440, PRO POWER, $28.99 at Newark.com for 300 ft)
  - 5 min Epoxy (14250, Devcon, $10.35 at Grainger.com)
  - 15 min Black Plastic Bonder (50139, J-B Weld, $6.99 at Amazon.com)
  - (optional) Shrink tubing (HS101-1/16, Insultab, $3.03 at McMaster-Carr, Item 7496K82)
  - Connector receptacle 3 position (22-01-3037, MOLEX, $0.08 at Newark.com
  - Connector receptacle 2 position (22-01-3027, MOLEX, $0.28 at Newark.com)
  - Connector headers 3 position (22-27-2031, MOLEX, $0.19 at Newark.com)
  - Connector headers 2 position (22-23-2021, MOLEX, $0.18 at Newark.com)
  - Contact sockets (08-50-0032, MOLEX, $0.18 at Newark.com)
  - 10 ml syringe (5100-X00V0, Henke-Ject, Henke Sass Wolf, Germany, $35.91 at VWR.com)
  - 1 ml syringe (309659, BD, $32.97 at VWR.com for 200)
  - Screw (3/16 in or 4.6 mm thread diameter, at least 5 cm long).
  - (optional) tubing (Tygon 3350 1/16 x 3/32, St. Gobain, $133 for 25 ft at Grainger.com)
  - (optional) matte tape (Magic Tape, Scotch, $3.50 at Amazon.com)

The tube holder consists of a cut-off 10 ml syringe fitted with a perpendicular 1 ml syringe as LED and photosensor holders. The syringe plungers were removed and the tips of the syringes cut off with a razor blade. Note that any comparable transparent vessel holding the desired culture tube to be measured would work.


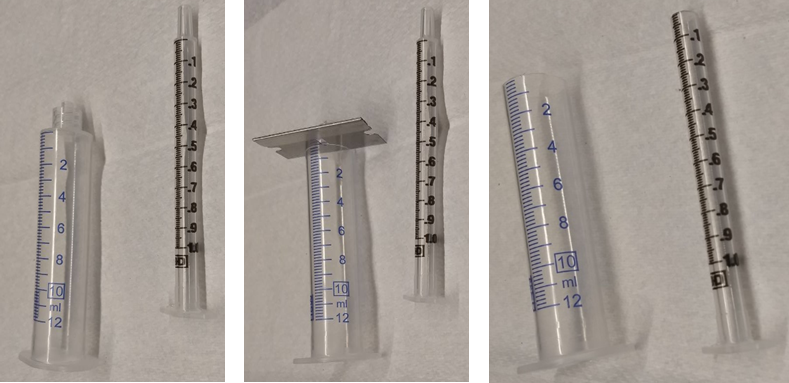


*Supplementary Figure 13. the plunger was removed from a 12 ml and a 1 ml syringe (left panel) and the tips of the syringes cut off with a razor blade (middle panel). The open tops of the syringes (right panel) fit now exactly around a Hungate tube (12 ml syringe) and a 5 mm LED (1 ml syringe).*

A 6.5 mm hole was drilled trough the 12 ml syringe roughly at the 12 ml mark. The height of the hole from the bottom of the syringe determines at which distance from the bottom of the tube the OD reading is performed. The hole has to be above the curvature of the bottom of the tube, but below the fill level of medium in the tube. The fit and centering of the 1 ml syringe through the larger syringe was checked by visual inspection. The 1 ml syringe was subsequently cut with a cork borer of the same diameter as the 10 ml syringe to obtain 2 pieces that fit perfectly onto the outside of the larger syringe.


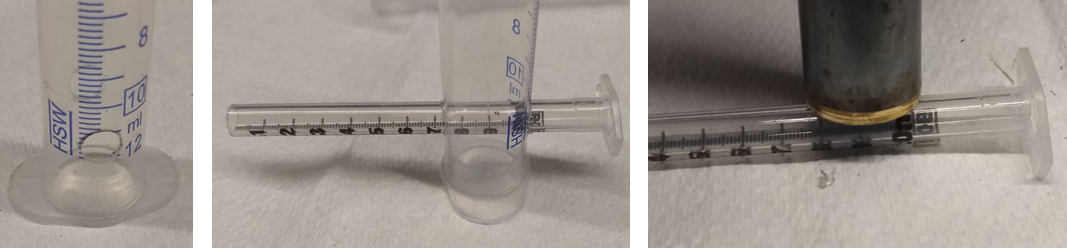


*Supplementary Figure 14. Two holes were drilled through the 12 ml syringe at the 12 ml mark (left panel). The 1 ml syringe was inserted completely through the hole to make sure the holes opposite to each other and the 1 ml is centered through the 12 ml syringe and to estimate where to cut the 1 ml syringe (middle panel). Then, the 1 ml syringe was cut with a cork borer to obtain 2 pieces whose inner edge has the same curvature as the 12 ml syringe (right panel).*

The size of the tip-part of the 1 ml syringe was shortened to about 1.5-2 cm. Then, a screw (which can be replaced by anything that fits tightly into the 1 ml syringe) was inserted through the pieces as seen in Figure 14 (middle) to hold them in place and ensure a straight light path through the assembly. The assembly is then glued together with a generous amount of 5-min epoxy.


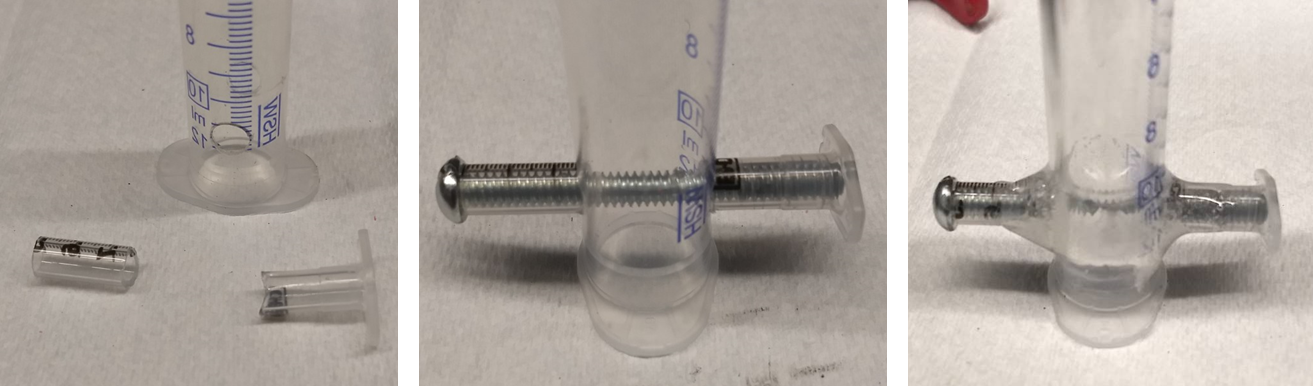


*Supplementary Figure 15. The tip part of the 1 ml syringe was shortened to about 1.5-2 cm (left panel). A 5 mm diameter screw was used to align the 2 pieces of the 1 ml syringe to the holes in the 12 ml syringe (middle panel). Leaving the screw in place, the assembly was glued in place with a liberal amount of 5 minute epoxy taking care not to glue the screw onto the syringes (right panel).*

The photosensor was soldered to the 3-pin connector header delivered with the sensor and the pins bent at a 90 degree angle to be parallel with the sensor. The LED –LED driver assembly was prepared as described above for the ClampOD and soldered to a 2-position connector header. The photosensor was then glued with black plastic bonder to the bottom side and the LED inserted into the tip side of the 1 ml syringe.


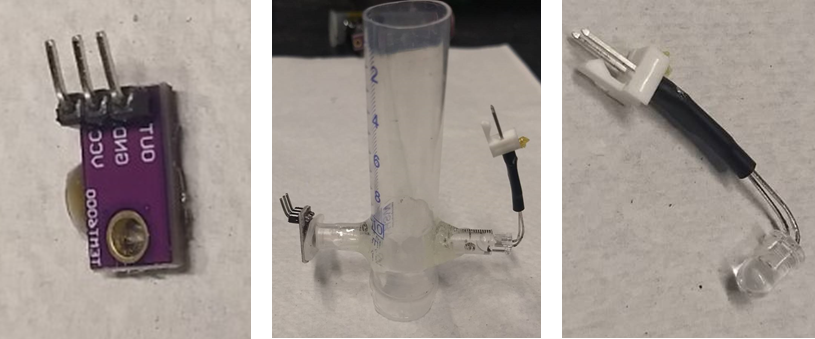


*Supplementary Figure 16. A 3 pin connection header was soldered to the photosensor and the connection pins bent to be parallel to the photosensor chip (left panel). The LED assembly was soldered to a pin connection header and the connections coated in glue and covered with heat shrink tubing (right panel). The photosensor was glued with black plastic bonder to the bottom side of the 1 ml syringe, and the LED inserted into the cut-off tip side (middle panel).*

The sensor side of the assembly should also be completely coated with black glue to limit the interference of ambient light. Optionally, the entire construct can then be coated with the black plastic bonder to further limit the effect of ambient light.


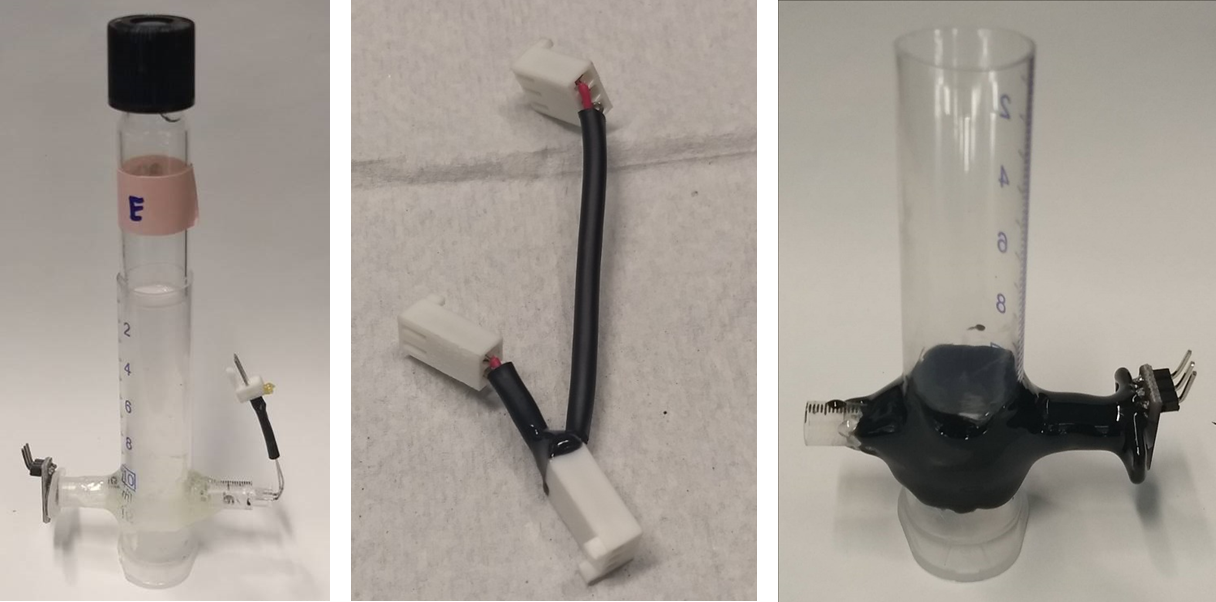


*Supplementary Figure 17. The assembled photometer fits exactly around a standard Hungate tube (e.g. CLS-4208, chemglass) (left panel). A short connector according to the wiring shown in the main manuscript and described for the ClampOD was prepared to connect each TubeOD to a longer cable to connect to the data acquisition system (middle panel). The light path between LED and Photosensor was coated liberally in black plastic bonder to minimize the effect of ambient light on the voltage readings (right panel).*

If the light intensity reaching the photosensor is exceeding its linear measurement range, a piece of tubing can be inserted into the 1 ml syringe piece housing the LED. The length of the tubing should be chosen so that a blank, medium filled tube results in a maximum voltage output of the photosensor of about 90% of the maximum voltage. For example, with a 3V power supply, the maximum output should be about 3V. Therefore, the target output with the inserted blank tube should be around 2.7 V.


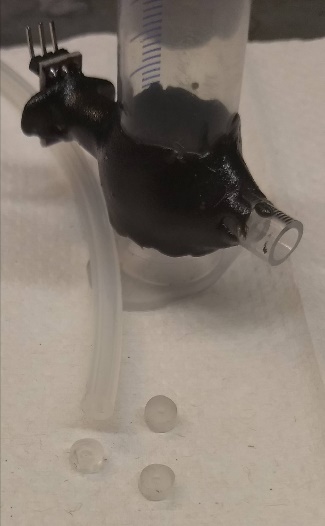

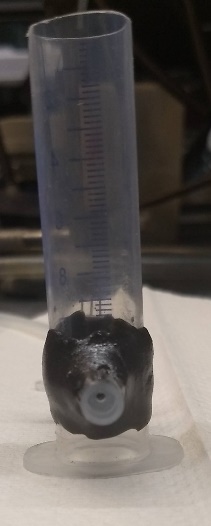


*Supplementary Figure 18. to attenuate the incident light intensity, pieces of tubing are cut (left panel) and inserted into the 1 ml syringe piece (right panel) housing the LED. Longer pieces of tubing will cause stronger light attenuation.*

## Data analysis:

Voltage and Ultraspec2100 OD data was analyzed in MATLAB (ver. R2021a) using the fit function with 1st and 2nd order fits. Residuals were checked visually for normality with the histfit function and the root mean squared error was calculated for each calibration. The link to the MATLAB code and raw data to generate the figures are available here: https://github.com/gcallander/OnlineOD

.
